# Defining the Enterovirus Diversity Landscape of a Fecal Sample: A Methodological Challenge?

**DOI:** 10.3390/v8010018

**Published:** 2016-01-12

**Authors:** Temitope Oluwasegun Cephas Faleye, Moses Olubusuyi Adewumi, Johnson Adekunle Adeniji

**Affiliations:** 1 Department of Microbiology, Faculty of Science, Ekiti State University, Ado Ekiti, Ekiti State, Nigeria; faleyetemitope@gmail.com or faleyetope@yahoo.com; 2 Department of Virology, College of Medicine, University of Ibadan, Ibadan, Oyo State, Nigeria; adewumi1@hotmail.com; 3 WHO National Polio Laboratory, Department of Virology, College of Medicine, University of Ibadan, Ibadan, Oyo State, Nigeria

**Keywords:** enteroviruses, enterovirus diversity landscape, cell culture, species bias

## Abstract

Enteroviruses are a group of over 250 naked icosahedral virus serotypes that have been associated with clinical conditions that range from intrauterine enterovirus transmission withfataloutcome through encephalitis and meningitis, to paralysis. Classically, enterovirus detection was done by assaying for the development of the classic enterovirus-specific cytopathic effect in cell culture. Subsequently, the isolates were historically identified by a neutralization assay. More recently, identification has been done by reverse transcriptase-polymerase chain reaction (RT-PCR). However, in recent times, there is a move towards direct detection and identification of enteroviruses from clinical samples using the cell culture-independent RT semi-nested PCR (RT-snPCR) assay. This RT-snPCR procedure amplifies the *VP1* gene, which is then sequenced and used for identification. However, while cell culture-based strategies tend to show a preponderance of certain enterovirus species depending on the cell lines included in the isolation protocol, the RT-snPCR strategies tilt in a different direction. Consequently, it is becoming apparent that the diversity observed in certain enterovirus species, e.g., enterovirus species B(EV-B), might not be because they are the most evolutionarily successful. Rather, it might stem from cell line-specific bias accumulated over several years of use of the cell culture-dependent isolation protocols. Furthermore, it might also be a reflection of the impact of the relative genome concentration on the result of pan-enterovirus *VP1* RT-snPCR screens used during the identification of cell culture isolates. This review highlights the impact of these two processes on the current diversity landscape of enteroviruses and the need to re-assess enterovirus detection and identification algorithms in a bid to better balance our understanding of the enterovirus diversity landscape.

## 1. Introduction

Enteroviruses (EVs) are a group of over 250 naked icosahedral virus serotypes with a diameter of 28–30 nm that are categorized as members of the genus *Enterovirus* in the family *Picornaviridae,* order *Picornavirales*. Currently, there are 12 species within the *Enterovirus* genus, out of which seven (enterovirus A–D (EV-A–EV-D) and human rhinovirus ((HRV)A–C) have been associated with human infection and disease [1]. Within the non-enveloped icosahedral capsid of an enterovirus is a protein-linked, single-stranded, positive-sense, ~7.5 kb RNA genome, which has a single open reading frame (ORF). The ORF is flanked on both sides (5′ and 3′ ends) by untranslated regions (UTRs) and translated into an ~250-kDa polyprotein. This polyprotein is auto-catalytically cleaved into P1, P2 and P3 polyproteins, which are further cleaved into VP1–VP4, 2A–2C and 3A–3D, respectively. The capsid is formed by VP1–VP4, with VP1, VP2 and VP3 exposed on the virion outer surface, while VP4 is buried within the virion. The other seven polyproteins (2A–2C and 3A–3D), are nonstructural proteins and are crucial in enterovirus replication.

Enteroviruses have been associated with a host of clinical conditions, which include intrauterine enterovirus transmission with fatal outcome [2], encephalitis, meningitis, pleurodynia, herpangina, conjunctivitis, gastroenteritis, myopericarditis, pancreatitis, hepatitis, type 1 diabetes, hand, foot and mouth disease, upper and lower respiratory tract diseases and paralysis or myelitis [3]. Besides the association of different enterovirus types with the same clinical manifestation, the same enterovirus type has also been associated with different clinical manifestations. Furthermore, because >90% of enterovirus infections are asymptomatic [4], most of the infections with clinical manifestation represent less than 10% of enterovirus infections [4]. In fact, in the United States alone, about 10–15 million enterovirus infections have been estimated to occur annually [5]. Enteroviruses are transmitted mainly via the fecal-oral route. However, transmission via the respiratory route and through conjunctival fluid have also been documented [4]. However, the Global Polio Laboratory Network (GPLN), which contains over 150 laboratories globally and is the largest repository or producer of information on enteroviruses, mainly investigates fecal specimens. Hence, this review will particularly address the enterovirus diversity landscape question with particular focus on experience gathered analyzing fecal specimens.

## 2. History of Enterovirus Classification

### 2.1. Initial Isolation and Classification Strategies

The study of enteroviruses started as a result of the dreaded disease poliomyelitis. Though known and dreaded for its paralysis-causing ability, it was not until 1908, when it was shown to be a “filterable agent” [6], that the concept of poliovirus (PV) started taking shape. Subsequent to its isolation in tissue culture and the demonstration of its serological types [7], poliovirus started off the field of enterovirology. This happened because, in addition to polioviruses, other enteric viruses were present in the feces of children with “paralytic disease” indistinguishable from poliomyelitis. These other viruses include coxsackievirus A (CV-A) and B (CV-B), echoviruses (Es) and the numbered enteroviruses. These viruses were isolated and identified using a combination of histopathology in newborn mice, cytopathic effect (CPE) in cell culture and serology [8,9,10].

### 2.2. Identifying the Value of VP1 in Enterovirus Identification

In the early days of enterovirology, isolate identification was done by neutralization assays, which prevented the development of the enterovirus-specific cytopathic effect in cell culture. It later became clear that antibodies elicited against the VP1 protein had neutralizing activity [11,12]. Subsequently, the binding sites of these neutralizing antibodies were localized to specific epitopes in the protein product of the *VP1* gene [13,14,15,16].

### 2.3. Equating Serotypes and Genotypes

The confluence of four things ushered in the era of enterovirus molecular identification: (1) the existence of previously-neutralized and identified pure cultures of enterovirus reference strains; (2) knowing that the *VP1* gene was largely responsible for defining enterovirus serotype; (3) the mainstreaming of primer synthesis and polymerase chain reaction (PCR); and (4) automation of Sanger sequencing. With all the above in place, Oberste *et al.* [17] showed the association between *VP1* sequence data and enterovirus serotypes. This was independently confirmed by several investigators using both previously-neutralized pure cultures and field strains [17,18,19,20,21,22,23,24,25]. Consequently, enterovirus identification became synonymous with *VP1* amplification and sequencing (molecular identification).

Prior to molecular identification, as previously mentioned, enteroviruses were classified along historical lines as PVs, CV-A and CV-B, Es and numbered EVs. However, molecular identification and the consequent phylogenetic analysis showed that “human” enteroviruses could be unequivocally classified into four (4) different species (EV-A–EV-D). This further resulted in the incorporation of polioviruses into the EV-C and the reclassification of former CV-A15, CV-A18, HRV-87 and swine vesicular disease virus (SVDV) to CV-A11, CV-A13, EV-D68 and CV-B5, respectively [26]. At the time of writing, EV-A contained 25 serotypes made up of some CV-As and some numbered enteroviruses. EV-B contained 63 serotypes consisting of CV-A and CV-B, the echoviruses and some numbered enteroviruses. EV-C contained 23 serotypes consisting of the remaining CV-As, the three poliovirus serotypes and some numbered enteroviruses. EV-D contained five serotypes consisting only of numbered enteroviruses [1].

### 2.4. Identifying the “Untypables”

**Figure 1:**
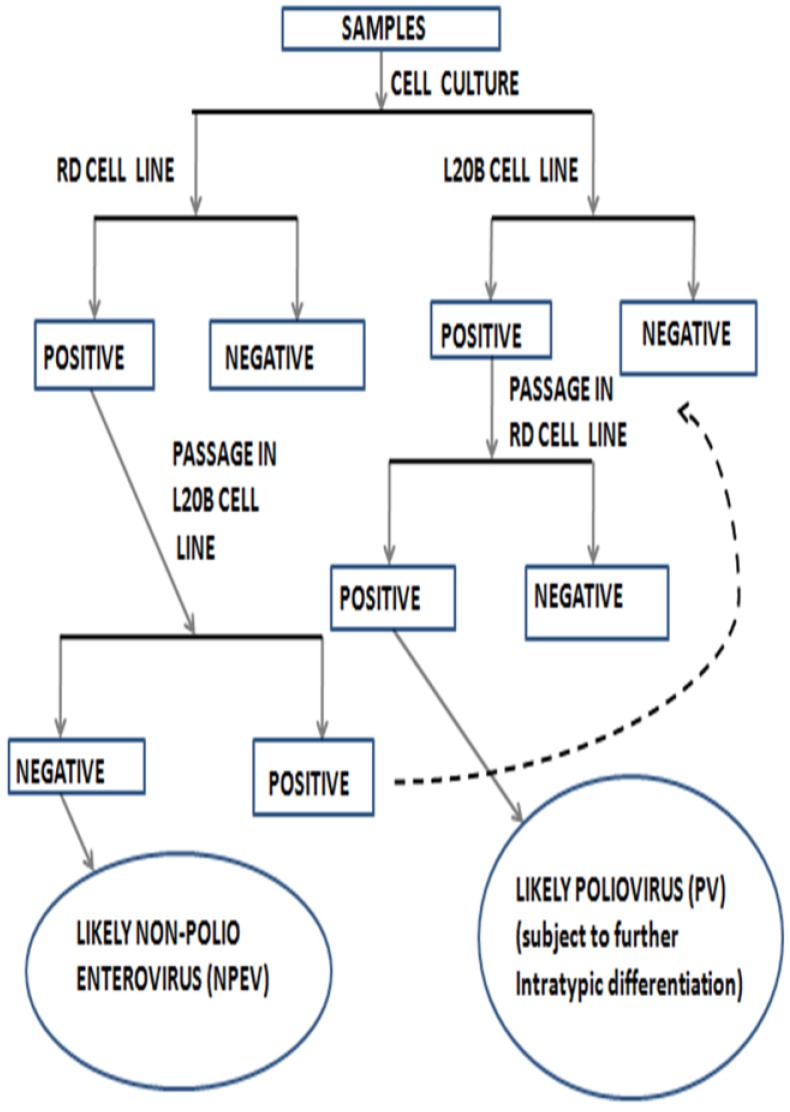
Poliovirus (PV) isolation algorithm as recommended by the World Health Organization (2003, 2004).

The Global Polio Eradication Initiative (GPEI) started as a result of the World Health Assembly’s (WHA) resolution in 1988 to eradicate poliomyelitis. A very strong arm of the eradication effort has been surveillance. However, a by-product of poliovirus surveillance has been an array of non-polio enteroviruses (NPEVs) (Figure 1). Prior to the development of enterovirus molecular identification, the types of most of these NPEVs were unknown because the then available panel of antisera could only identify 40 of the first 66 enteroviruses that were characterized [27]. This left an array of unidentified enteroviruses referred to as the “untypables”. With molecular identification, many of these previously “untypable” enterovirus isolates have been identified and new types discovered.

## 3. Impact of Cell Line Bias on the Enterovirus Diversity Landscape

Identification of previously “untypable” NPEV isolates further increased the preponderance of EV-Bs. This strengthened the impression that EV-Bs were the most diverse and further enhanced an enterovirus diversity picture that is skewed in the EV-B direction. It is however becoming apparent that the diversity observed in EV-Bs might not be because they are the most evolutionarily successful. Rather, it might stem from two different factors. Firstly, some enteroviruses cannot, currently, be isolated in cell culture [26], and secondly, the RD cell line used by the GPLN [28], appears to preferentially support the replication of EV-Bs even in the presence of members of other enterovirus species [29,30]. By including other cell lines (e.g.,Hep 2C and MCF-7) in enterovirus isolation protocols, the rate at which EV-C members are detected has increased [31,32]. For example, in most cases, when the same sample is simultaneously inoculated into RD and MCF-7 cell lines, both will specifically isolate EV-Bs and EV-Cs, respectively [30].

In cases were both EV-C and EV-B are present in an isolate recovered on the RD cell line, in our laboratory, we have observed that direct molecular identification without first separating the mixture usually selectively identifies the EV-B component of the mixed isolate [29]. Against this backdrop of the RD cell line EV-B bias, it appears as though something in the biology of the cell line selectively supports the replication of EV-Bs. This might result in increased titer/relative genome concentration, which is later magnified by molecular identification. If relative genomic concentration significantly impacts molecular identification, how then does this translate into direct molecular identification of enterovirus from clinical specimens.

## 4. Molecular Identification without Cell Culture

**Figure 2:**
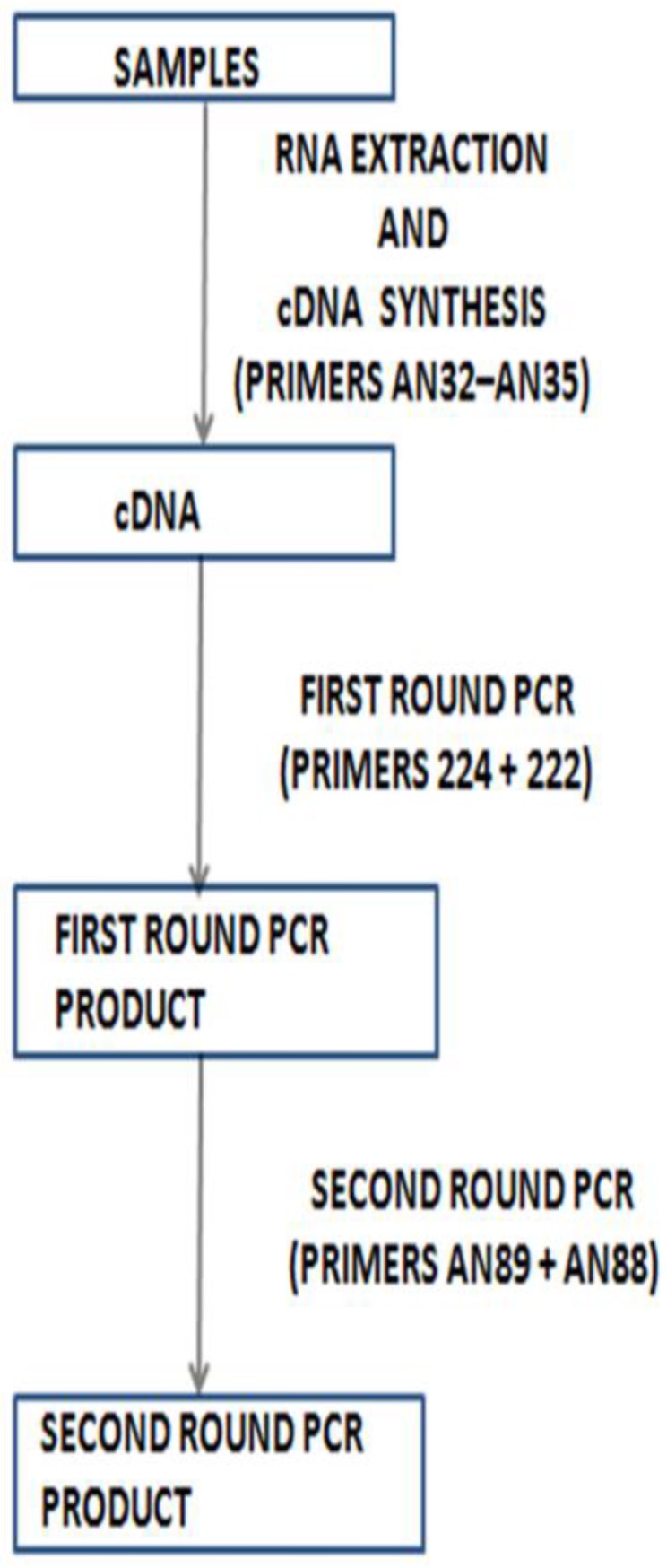
Recently recommended algorithm for direct detection of enteroviruses from clinical specimens (WHO, 2015).

One strategy that has been proposed to overcome the impact of cell culture bias is direct molecular identification of enterovirus from clinical specimens using a reverse transcriptase semi-nested PCR (RT-snPCR) assay (Figure 2), and this has been documented to work [33]. This strategy has now been adopted officially by the World Health Organization (WHO) [34] as an appendage to its previous cell culture-based strategy for enterovirus surveillance [35,36]. Considering the preponderance of enterovirus co-infections, should the genomic concentration have any impact on the final identity of mixed enteroviruses, then the enterovirus with the highest titer in clinical samples might consistently be identified. This phenomenon of mixed isolates bearing the identity of only one member of the mixture has recently been documented [37,29]. In fact, we have recently found that when the Nix *et al.* protocol is applied directly to clinical samples, enterovirus isolates that cannot be detected can subsequently be detected after the samples are first subjected to cell culture using a susceptible and permissive cell line [38]. This confirms that genomic concentration significantly impacts the enterovirus serotype detected by the Nix *et al.* protocol.

**Figure 3:**
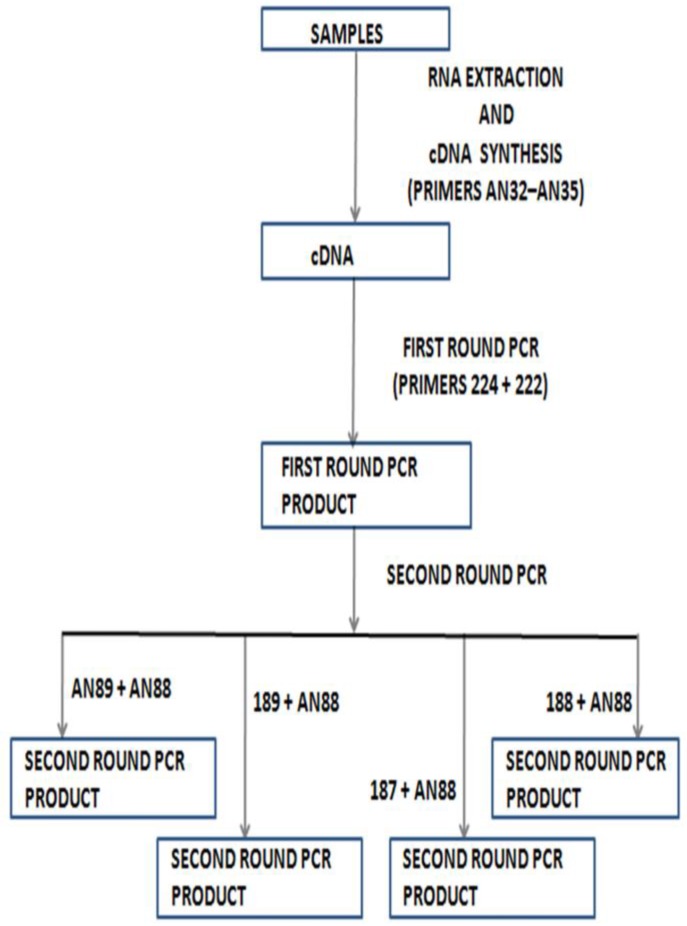
Modified WHO algorithm used in our laboratory for direct detection of enteroviruses from clinical specimens.

This shows that as much as molecular identification enables us to find and identify enteroviruses that would have been undetectable, an attempt to leave the cell culture-based algorithm behind, if not carefully managed, will also bias our view of the enterovirus diversity landscape. One of the ways in which we have tried to address this problem is by adding a species-specific screen using primers 187, 188 and 189 [20,34] to the second round PCR of the Nix *et al.* protocol [33]. Hence, instead of one second round PCR, there were four different second round PCR assays that use the same first round PCR product as a template (Figure 3). When this strategy was used to screen fecal samples and RD cell line isolates, either paired or not, we found a number of interesting things [38,39].

Firstly, it was noticed that the Nix *et al.* protocol, though very sensitive for detecting enterovirus genomes, would often mask the presence of more than one enterovirus isolate per sample [39]. The phenomenon was however inherited from primers 292 and 222 [29], which were upgraded into AN89 and AN88 [33]. However, primer 292 (and by extension AN89) is a consensus of primers 187, 188 and 189 [20], and the inclusion of these three primers restored the resolving power of the assay [39]. Hence, by modifying the WHO recommended [34] Nix *et al.* protocol [33] to independently use primers AN89, 187, 188 and 189 (forward primers) alongside AN88 (reverse primer) for the second round PCR, the assay retains its sensitivity for enterovirus detection and is now more valuable for identification due to its mixed isolate-resolving capacity [39].

Secondly, it was noticed that whatever isolate shows up on the RD cell line is usually not the complete picture. Most of the time, other enteroviruses are present in the sample that will not grow on the RD cell line [38]. Therefore, studies based on the RD cell line (or others with their different biases unaccounted for) that have associated certain enterovirus strains to specific clinical conditions [40,41] should be interpreted with caution, because the likelihood exists that not all of the enteroviruses in the sample were detected. Therefore, considering that most enterovirus infections are asymptomatic and we hardly truly exhaustively catalogue the enterovirus diversity landscape of a sample, it is difficult to conclude which enterovirus or combination of enterovirus types are really associated with the clinical manifestation.

Thirdly, even when a clinical sample is negative for enteroviruses by the pan-enterovirus *VP1* screen based on primers 224, 222, AN89 and AN88 (Nix *et al.*, 2006, protocol) [33], usually, the species-specific screen (using the first round product of 224 and 222 as a template) still detects, at times, about two different serotypes in the same sample. Finally, as previously mentioned, an enterovirus serotype that was not detected in the clinical specimen even after repeated screening will show up after cell culture. This suggests that the pan-enterovirus *VP1* screen (Nix *et al.*, 2006, protocol) [33] is not infallible and virus concentration significantly impacts the detectability of virions present in samples. Consequently, the importance of susceptible and permissive cell lines and “mixture”-resolving primers cannot be overemphasized. We should therefore be careful about declaring a sample negative for enteroviruses.

## 5. Shortcomings of Molecular Identification

One of the issues that molecular identification does not totally cater to is the fact that the serotype of any virus is also a phenotype. Like all phenotypic properties, serotypes are subject to selective pressures and consequently evolve and ensure survival of the serotype. For example, studies [42,43] have shown that antibodies elicited by older strains of a particular serotype are usually not as effective in neutralizing modern strains of the same serotype. These isolates are usually referred to as immune escape mutants (IEMs) [28]. This phenomenon has been suggested [28] to be responsible for the recent wild poliovirus (WPV) 1 outbreaks in the Republic of Congo [44] and Israel [46]. How then do we identify the first occurrence of IEMs from molecular identification data in the absence of experiments to associate the phenotype with specific mutations?

Another issue is the existence of dual-serotype-specific (DSS) enterovirus isolates [46,47,48]. The DSS enterovirus isolate is one that is simultaneously neutralized by antibodies elicited against two independent enterovirus serotypes. The place of isolates with such a phenotype in the evolutionary biology of enteroviruses is currently unknown and unaccounted for by molecular identification, which ascribes one unequivocal serotype to an enterovirus isolate.

## 6. Serotype is Not the Full Picture: Same Serotype, Different Phenotypes/Biological Properties

However, as important as determining the serotype of an enterovirus might be in the identification of enteroviruses, it seems not to be the full picture. It is common knowledge in enterovirology that little or no association exists between enterovirus serotypes and clinical manifestations [49]. Hence, from a clinical perspective, is the serotype the best way to define enterovirus isolates or the diversity landscape?

Considering the high recombination frequency of enterovirus genomes [50], one can hardly find a cluster of enteroviruses of the same serotype that are truly the same in all genomic regions. In fact, studies [51,52,53,54,55,56,57,58] have shown that the difference in the transmissibility and pathogenicity between two revertant Sabin-like polioviruses might be as simple as the origin of the non-structural region they each possess. Hence, *VP1* phylogeny does not imply much with respect to other genomic regions or biological properties of enteroviruses [59].

Besides clinical manifestation, another dimension to the enterovirus diversity landscape is in their receptor usage. The prevailing paradigm is that a particular enterovirus serotype uses one or a defined set of receptors (and co-receptors). Studies have now shown that this picture might also not be complete. As a starter, it was recently shown that CD150 is not the only cell surface receptor for measles virus [60]. In fact, it was shown that when being transferred from lymphocytes to epithelial cells, measles viruses use poliovirus receptor-like 4 (PVRL4) as their receptor to enter into epithelial cells [61,62]. In similar light, CV-A20 strains exist that can independently use either Intercellular Adhesion Molecule 1(ICAM 1) or another yet to be described cell surface molecule as the receptor [64]. More recently, Shimizu’s group hinted at the existence, in feces of poliomyelitis cases, poliovirus strains that do not bind the poliovirus receptor (PVR) [37,64]. They consequently describe these strains as “non-infectious”. However, though these PV strains might not be detected by the current poliovirus isolation protocol, which is rigged to detect strains that use PVR as their cognate receptor, it cannot be concluded unequivocally that these PV strains are “non-infectious” in the field, since they were detected in multiple samples. Considering that it has been shown that PVR might not be the receptor polioviruses use to enter into neurons [65], there might still exist at least a second poliovirus receptor. The observations of Shimizu’s group might be anecdotal evidence that a similar phenomenon might be ongoing with polioviruses [37,64].

## 7. Conclusion: Enterovirus Diversity Landscape, What Is Really out There?

What if the phenomenon of the association of different enterovirus serotypes with the same clinical manifestation and *vice-versa* is just a reflection of the fact that over history, we have not been able to really fully catalogue every enterovirus isolate present in any sample. Furthermore, what if being able to fully catalogue the enterovirus diversity landscape in every sample and population will show us the co-operative effects of simultaneous infection by different enterovirus serotypes and its influence on clinical manifestations?

We are already in the early parts of the second century of enterovirus research. It is only proper that going forward, effort should also be focused on developing techniques or a combination of techniques that are very sensitive and highly specific for enterovirus detection in fecal and other specimens (e.g., respiratory) that might contain the virus. These techniques should enable us to better detect and identify enteroviruses when present and to properly resolve members of different species and, more specifically, members of the same species present in the same sample. Technologies like digital PCR, Next-Generation Sequencing (NGS) using enterovirus specific libraries and a merger of these with cell culture might help accomplish the stated. Our ability to accomplish this will enable us to better characterize the plethora of enterovirus serotypes present in any given sample and/or simultaneously circulating in a population at any point in time. Our ability to effectively do this might shed light on the enigmas that currently exist in the field of enterovirology and better inform us on what is really out there.
